# Growth Performance of Rabbits Fed Palm-Press Fibres-Based Diets

**DOI:** 10.5402/2012/915729

**Published:** 2012-07-09

**Authors:** M. Frederic Houndonougbo, C. A. A. M. Chrysostome, S. E. Attakpa, A. Sezan, H. B. Dehou

**Affiliations:** ^1^Faculty of Agronomic Sciences, University of Abomey Calavi, 01 BP 526 Cotonou, Benin; ^2^Faculty of Technical Sciences, University of Abomey Calavi, 01 BP 4521 Cotonou, Benin

## Abstract

An experiment was carried out to define the optimal rate of palm-press fibres in growing rabbits' diet. In total, 64 weaned rabbits (35 days old) of Beninese breed were divided in 16 groups of 4 rabbits (2 males and 2 females) each. During six weeks, rabbits were fed with 4 complete diets containing 0% (F0, control), 5% (F5), 10% (F10), and 15% (F15) of fibres from a palm oil industry. Results demonstrated that up to 15 of palm-press fibres can be included efficiently in growing rabbits' diet. The daily feed intake was not significantly affected by the diet (*P* > 0.05). At 13 weeks old, the average live weights of rabbits were 1788.5 g, 1805.0 g, 1718.5 g, and 1801.3 g in respectively, F0, F5, F10 and F15 groups. No mortality of rabbits was recorded. Compared to F0, the feed conversion ratio and feeding cost decreased in the group of rabbits fed F15 diet. The carcass yield was similar between diets.

## 1. Introduction

In Benin, 64% of the population has consumed once rabbit meat at least, and 95% of them appreciated it positively [1]. Thus, to supply the market with white meat, rabbits' farms are installed in periurban areas. To promote optimal growth performance of rabbits, appropriate feeding strategy is necessary. Fibres are one of the main components of rabbits' diets [2]; because they play a key role in rabbit feeding by contributing to caecum activity for efficient digestion [3]. Moreover, [2] demonstrated that particle size is a major factor of fibre digestibility in rabbits. Crude fibre level in growing rabbits' diet varies from 14 to 16%, whereas in reproductive rabbits' diets that level is between 12 and 13% [4].

Unfortunately, the availability of forages, main sources of fibre is low in peri-urban areas, and farmers have difficulties to provide rabbits with grass. The processing of complete diets with optimal level of fibres is therefore suitable to efficiently feed rabbits. Such feeding approach can be done by using different low cost and available feedstuffs. Palm-press fibres are available in many palm oil industries in Benin. They are extracted from the oil palm empty fruit bunches around the nut. Palm-press fibres contain about 86.2% of dry mater, 4% of crude proteins, 21% of fat, 0.31% of Calcium, 0.13% of Phosphate and 36.4% of crude fibre [5]. Palm-press fibres can be dried and pelleted to overcome the problems of poor keeping quality and bulkiness [6]. They can be used in high fibre breads [5]. Palm-press fibres can be therefore included in complete diets of rabbits, rodents and ruminants. Crude proteins and crude fibre digestibility decreases when the level of palm-press fibres exceeds 25–30% in ruminants diet [6]. The marketing of palm-press fibres for animal feeding will provide additional revenues to palm oil producers in developing countries.

The objective of this study was to contribute to the valorization of palm-press fibres in growing rabbits feeding by evaluating its optimal level in diets.

## 2. Materials and Method

### 2.1. Animals and Housing

A total of 64 rabbits weaned at 35 days old were divided in 16 groups of 4 rabbits each. At the beginning of the experiment, the average live body weight of these rabbits of local breed reared in Benin was 857.2 g. 

 Each group of 4 rabbits (2 males and 2 females) was housed in a cage (length × width × height : 80 × 50 × 30 cm^3^). Cages were installed in two rows of 8 cages each. They were at 70 cm from the ground and 180 cm from the roof. Each cage had a drinker and two feeders.

### 2.2. Experimental Feeds and Design

Four experimental feeds were used. They contained, respectively, 0% (F0, control diet), 5% (F5), 10% (F10), and 15% (F15) of palm press fibres (Table 1). Palm press fibres were from a palm oil industry. Feeds were in meal. They had similar digestible energy and crude proteins (Table 2). Rabbits were fed only with the experimental feeds without any addition of forage. 

Each feed was delivered to four cages of four rabbits each. Per feed, two cages of rabbits were randomly selected in each row. At the beginning of the experiment, similar (*P* > 0.05) average live body weights of rabbits were recorded in F0 (858.1 g), F5 (836.3 g), F10 (880.0 g), and F15 (854.4 g) dietary treatments. The experiment took six weeks and finished when rabbits were thirteen weeks old.

At six weeks old, four starved rabbits (2 males and 2 female) were slaughtered per diet for carcass study. The full digestive tract, carcass, liver, and abdominal fat were weighted. Relative weights of liver and full digestive tract were calculated on carcass weight basis.

### 2.3. Statistical Analysis

Data were analyzed using general linear model (GLM) in SAS version 9.1.2 [7]. The performances of rabbits were compared using each cage of four rabbits (two males and two females) as repetition. Repetition effect and interaction between diets and repetitions were not significant (*P* > 0.05). Thus, analyses were performed according to the model as follows:

(1) $$\begin{array}{c} Y_{i}=\mu +F_{i}+\varepsilon_{i}, \end{array}$$

where *Y*_*i*_ is the observation for dependent variables; *μ* is the general mean; *F*_*i*_ is the fixed effect of the feed; *ε*_*i*_ is the residual error. 

## 3. Results 

### 3.1. Feed Intake of Growing Rabbits

**Figure 1:**
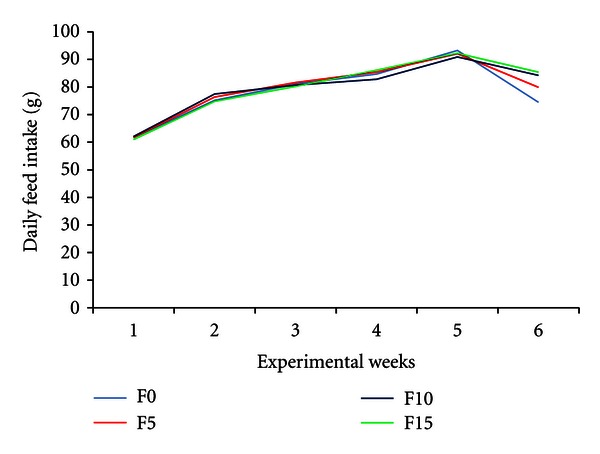
Average daily feed intake of growing rabbits fed palm-press fibres-based diets. F0, F5, F10 and F15 are diets containing 0, 5, 10, and 15% of fibres respectively.

The daily feed intakes of rabbits fed with the four diets (Figure 1) were similar during the experiment (*P* > 0.05). On average, daily feed intakes were, respectively, 78.4 ± 10.7 g (F0), 79.5 ± 10.2 g (F5), 79.7 ± 9.7 g (F10) and 79.94 ± 11.0 g (F15). In the sixth week of experiment (13 week old) the feed intake decreased in all dietary treatments. The decrease of feed intake was more important when the level of crude fibre in diet is low as it was the case in F0 and F5 diets (Table 2). 

### 3.2. Growth Performance of Rabbits and Efficiency of Feeds

**Figure 2:**
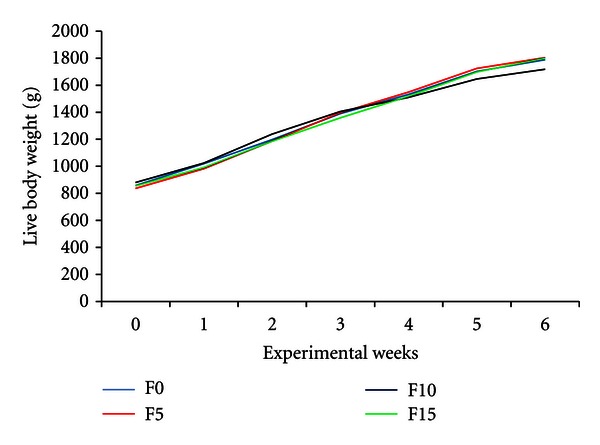
Growth curves of rabbits fed palm-press fibres-based diets. F0, F5, F10 and F15 are diets containing 0, 5, 10, and 15% of fibres, respectively.

The growth of rabbits was regular (Figure 2). The daily body weight gains (Table 3) were similar between diets (*P* > 0.05). Thus, at the end of the experiment (77 days-old), the live body weights were not significantly different (*P* > 0.05) between rabbits fed F0 (1788.5 g), F5 (1805.0 g), F10 (1718.5 g) and F15 (1801.3 g).

The lowest feed conversion ratio and feeding cost were recorded in rabbits fed F15 diet (Table 3). The economic feed efficiency that reports the revenue from the live body weight gain comparatively to the feeding cost, was not significantly affected by the dietary treatment (*P* > 0.05). No mortality of rabbits was recorded during the study. Thus, included, up to 15% in balanced diet, palm-press fibres had no significant effect on the survivability of growing rabbits. Palm-press fibres-based diets were therefore efficient in growing rabbits feeding.

### 3.3. Carcass Characteristics

The carcass yields of rabbits were similar (*P* > 0.05) between dietary treatments (Table 4). Additionally, the relative weight of digestive tract and abdominal fat evaluated comparatively to the carcass weight were not significantly affected by the level of palm-press fibres in diets (*P* > 0.05). However, compared to the control diet (F0), the proportion of liver was significantly lower in rabbit fed F5 and F15 dietary treatments (*P* < 0.05). 

## 4. Discussion

### 4.1. Feed Intake of Growing Rabbits

The feed intakes recorded in all dietary treatments were close to 78.7 g/day [9]; but higher than 53.3–60.4 g/day [10] and lower than 136.3 and 149.9 g/j [11]. The light increase of feed intake of rabbits fed with palm press fibres-based diets (F5, F10 and F15) confirms the statements of [4, 12] according to which, the feed intake is positively correlated with the level of fibre in diet. In hot and humid climate, the keeping of an optimal level of feed intake is a challenge in livestock. The use of palm press fibres-based diets can therefore, reduce the requirement of forages to feed rabbits and allow them to develop their intestinal microflora that is necessary for efficient digestion.

### 4.2. Growth Performance of Rabbits and Efficiency of Feeds

The similar growth between rabbits fed different level of palm press fibres-based diets indicates the efficiency of that feedstuff in rabbit feeding. The daily weight gains of the experimental rabbits (19.65 to 23.07 g) were higher than the 7.48 to 10.91 g reported in Nigeria [10] and 16.7 g found in Benin with similar rabbit breed [13]. Compared to the control diet F0, F5 and F15 diets improved lightly the growth performance of rabbits without any adverse effect on their survivability. The final live body weights of rabbit (1718 to 1805 g) are close to 1746 g found at the same age in local population of rabbit in Tunisia [14]. The body weights of rabbits are also in the range of 1638.9 to 1862.5 g [15]. No medical prophylactic cure was provided to rabbits. The low performance of rabbits fed F10 diet might be due to a health or digestive problem. However, no external symptom was noticed. 

The decrease of feed conversion ratio and feeding cost in F5 and F15 diets confirms the efficiency of these diets and the profitability of palm press fibres inclusion in growing rabbits' diets. Feed conversion ratios (3.78 to 4.64 g feed/g body weight gain) were lower than 5.81 to 8.11 feed/g weight gain, found in Nigeria [10].

However, feeding costs (760 to 816 FCFA feed/kg body weight gain) were higher than about 596.4 to 685.1 FCFA feed/kg body weight gain [10]. This economic result can be explained by the feed price (189 to 192 FCFA/kg feed) that was higher than about 85.03 to 128.8 FCFA/kg feed reported by these authors. The result indicates in certain instance, the difference in feeds market between Benin and Nigeria. In monogastric livestock, feeding cost represents more than 60% of production cost. In broilers chickens, [16] reported 2.35 FCFA weight gain/FCFA feed as economic feed efficiency. Compared to broiler, growing rabbits fed palm fibres-based diets produce therefore, about equally profitable white meat in Benin.

### 4.3. Carcass Characteristics

The carcass yields (60.8 to 62.9%) are higher than 57.1% [17]; but lower than the 64.8 to 66.6% [18] reported in rabbit fed pellet diets and fodder. The palm press fibres-based diets did not affect significantly the relative weight of digestive tract of rabbits. The abdominal fat in rabbits carcass increase lightly in F5 and F15 diets compared to the control diet (F0). These results show a similar effect of the experimental diets on rabbit meat productivity and carcass quality. Moreover, the diets kept rabbits in apparently good health conditions as demonstrated the significantly low liver relative weight of rabbits fed F5 and F15.

## 5. Conclusion

This experiment suggests that growing rabbits can be fed exclusively with balanced palm press fibres-based diets. In such feeding strategy, up to 15% of palm press fibres can be included efficiently in the diets without any significant adverse effect on rabbits' survivability and their abdominal carcass fat. To reduce grass and forage need in peri-urban rabbitry, further investigations can be carried out on reproduction performance of rabbits.

## Figures and Tables

**Table 1:** Composition in ingredients and prices of growing rabbits' feeds as formulated and fed.

Ingredients (%)	F0	F5	F10	F15
Palm-press fibre	0	5	10	15
Palm-kernel meal	30	27	25	21
Maize grains	22	31	31	34
Wheat bran	28.6	14.6	9.6	2.6
Soybean meal	9	12	14	17
Cotton meal^1^	7	7	7	7
Oyster shell	1.7	1.7	1.7	1.7
Lysine	0.1	0.1	0.1	0.1
Methionine	0.1	0.1	0.1	0.1
Phosphate bicalcium	1	1	1	1
Salt (Nacl)	0.3	0.3	0.3	0.3
Premix^2^	0.2	0.2	0.2	0.2
Feed price (FCFA^3^/kg)	191.0	190.7	189.2	189.0

^1^Ferrous sulphate (FeSO_4_) were added at the rate of 3 g per kg of cotton meal.

^2^Premix contained per kg—vitamins: A 4000000 UI, D3 800000 UI, E 2000 mg, K 800 mg, B1 600 mg, B2 2000 mg, niacin 3600 mg, B6 1200 mg, B12 4 mg, choline chloride 80000 mg; minerals: Cu 8000 mg, Mn 64000 mg, Zn 40 000 mg, Fe 32000 mg, and Se 160 mg.

^3^Republic of Benin Currency: 1*€*= 655.9 FCFA.

**Table 2:** Chemical composition of growing rabbits' diets as formulated and fed.

Chemical components	F0	F5	F10	F15
Dry matter (%)	88.6	88.5	88.5	88.5
Total crude fibre (%)	9.34	9.79	11.0	11.8
Crude fibre from palm-press fibre (%)	0	1.82	3.64	5.46
Crude fibre from other ingredients (%)	9.34	7.97	7.33	6.35
Crude fat (%)	7.51	7.9	8.46	8.78
Digestible energy (kcal/kg)	2665	2667	2670	2675
Crude proteins (%)	17.62	17.4	17.5	17.6
Lysine (%)	0.82	0.83	0.9	0.88
Methionine (%)	0.38	0.38	0.4	0.38
Methionine + cystine (%)	0.71	0.70	0.7	0.69
Calcium (%)	1.01	1.00	1.0	1.02
Total phosphate (%)	0.94	0.79	0.7	0.66
Ca/P	1.07	1.27	1.4	1.55

**Table 3:** Daily weight gain (g), feed conversion ratio (g feed/g BWG^1^), feeding cost (FCFA^2^ feed/kg BWG), and economic feed efficiency (FCFA BWG/FCFA feed) of growing rabbits fed palm-press fibres-based diets.

	F0^3^	F5	F10	F15	SE^4^	*P* value
Daily weight gain	21.7 ± 6.4	23.1 ± 6.8	19.7 ± 6.5	22.5 ± 4.7	1.31	0.28
Feed conversion ratio	4.16 ± 2.0	3.95 ± 1.8	4.64 ± 2.1	3.78 ± 1.1	0.38	0.41
Feeding cost	816 ± 404	788 ± 368	949 ± 438	760 ± 231	76.6	0.35
Economic feed efficiency^5^	2.40 ± 0.8	2.46 ± 0.7	2.09 ± 0.8	2.38 ± 0.6	0.15	0.31

^1^Live body weight gain.

^2^Currency: 1€ = 655.9 FCFA.

^3^F0, F5, F10, and F15 are diets containing, 0, 5, 10, and 15% of palm-press fibres, respectively.

^4^SE: standard error.

^5^Revenue from 1 kg weight gain/feeding cost, [8].

**Table 4:** Carcass yield and organs' relative weight of growing rabbits fed palm-press fibres-based diets.

	F0^1^	F5	F10	F15	SE^2^	*P* value
Carcass yield (%)	62.9 ± 1.6	62.5 ± 2.4	60.8 ± 3.9	62.8 ± 1.4	1.26	0.604
Proportion full digestive tract (%)	20.2 ± 2.2	17.6 ± 2.1	19.4 ± 2.1	18.8 ± 1.9	1.039	0.410
Proportion of liver (%)	4.2^a^ ± 0.3	3.6^b^ ± 0.3	4.4^a^ ± 0.4	3.8^b^ ± 0.2	0.147	0. 009
Proportion of abdominal fat (%)	1.2 ± 0.9	1.6 ± 0.2	1.2 ± 0.5	1.5 ± 0.3	0.264	0.703

^1^F0, F5, F10, and F15 are diets containing, 0, 5, 10, and 15% of palm press fibres, respectively.

^2^SE: standard error.

^a, b^Means with unlike superscripts in the same rowdiffer significantly (*P* < 0.05).
